# State of the art in abdominal MRI structured reporting: a review

**DOI:** 10.1007/s00261-020-02744-8

**Published:** 2020-09-16

**Authors:** Arnaldo Stanzione, Francesca Boccadifuoco, Renato Cuocolo, Valeria Romeo, Pier Paolo Mainenti, Arturo Brunetti, Simone Maurea

**Affiliations:** 1 Department of Advanced Biomedical Sciences, University of Naples “Federico II”, Naples, Italy; 2 Department of Clinical Medicine and Surgery, University of Naples “Federico II”, Via S. Pansini, 5, 80131 Naples, Italy; 3 Institute of Biostructures and Bioimaging, National Research Council, Naples, Italy

**Keywords:** Structured report, Abdominal MRI, Evidence-based medicine, Report quality, Standardized lexicon

## Abstract

In the management of several abdominal disorders, magnetic resonance imaging (MRI) has the potential to significantly improve patient’s outcome due to its diagnostic accuracy leading to more appropriate treatment choice. However, its clinical value heavily relies on the quality and quantity of diagnostic information that radiologists manage to convey through their reports. To solve issues such as ambiguity and lack of comprehensiveness that can occur with conventional narrative reports, the adoption of structured reporting has been proposed. Using a checklist and standardized lexicon, structured reports are designed to increase clarity while assuring that all key imaging findings related to a specific disorder are included. Unfortunately, structured reports have their limitations too, such as risk of undue report simplification and poor template plasticity. Their adoption is also far from widespread, and probably the ideal balance between radiologist autonomy and report consistency of has yet to be found. In this article, we aimed to provide an overview of structured reporting proposals for abdominal MRI and of works assessing its value in comparison to conventional free-text reporting. While for several abdominal disorders there are structured templates that have been endorsed by scientific societies and their adoption might be beneficial, stronger evidence confirming their imperativeness and added value in terms of clinical practice is needed, especially regarding the improvement of patient outcome.

## Background

Allowing to evaluate both morphological and functional features of oncologic and non-oncologic diseases, magnetic resonance imaging (MRI) plays a major role in abdominal imaging. While a proper acquisition protocol and the correct identification and interpretation of key imaging findings are of uttermost importance to achieve optimal diagnostic accuracy, the quality of MRI reports should be considered equally crucial. Indeed, sentences that convey diagnostic uncertainty are frequently present in MRI reports, which are also often lacking in terms of clarity and extensiveness, thus leading to potential misunderstandings with the referring physicians [1, 2]. Therefore, the transition from narrative (NR) to structured reports (SR) has been considered a valid strategy to increase the value of diagnostic imaging [3]. The conventional NR are based on free-text and personalized language, characteristics that allow flexibility in reporting at the expense of variability in length, lexicon, style, and content [4, 5]. On the other hand, the main features of SR are format, content, and language standardization. More specifically, SR are organized into paragraphs with appropriate headings to identify essential sections (e.g., clinical setting and indication, imaging protocol, radiological findings, conclusions). Additionally, key imaging findings are arranged in a check-list style, which is contextualized and adapted based on the specific disorder/clinical indication, so that they are easily identifiable and cannot be mistakenly left out by the reporting radiologist. Finally, a standardized language must be embraced in SR, as it allows to minimize misinterpretations of pathological and normal findings and facilitate comparability of reports [2, 3, 6].

There are several studies suggesting that referring physicians prefer SR over NR as well as that SRs offer many advantages (e.g., fewer diagnostic error, higher consistency and comprehensiveness) leading to an overall improvement of report quality [2, 7, 8]. To promote the adoption of SR, the Radiological Society of North America offers a free and multilanguage radiology reporting template library (www.radreport.org). Similarly, the American College of Radiology provides a standardized framework for reporting on imaging findings (www.acr.org/Clinical-Resources/Reporting-and-Data-Systems). However, while scoring systems enhance MRI reporting through standardization, these can be used in NR as well as in SR and the concepts of standardized and structured reporting appear separate and complementary rather than redundant, with SR covering the creation of the report and its layout [9]. Furthermore, the transition from NR to SR is progressing slowly, and the use of SR is prevalent within subspecialty groups rather than widespread [10]. Finally, there are still radiologists who prefer NR as they are more accustomed to it, while others believe that SR could be limiting compared to their skill in fluent speech, diverting their focus from the images and reducing their efficiency [2, 8].

With this review we aimed to identify the main current applications of SR in abdominal MRI and discuss relevant evidence available in the scientific literature regarding its comparison to NR.

## Current applications

An overview of abdominal diseases for which structured MRI reporting recommendations released by scientific societies are presently available in the literature can be found in Table 1. Most of these recommendations are focused on oncologic disorders (e.g., renal masses, prostate cancer, endometrial cancer) and were drafted to meet a specific diagnostic need (e.g., lesion detection, staging). However, there are also some diseases for which SR templates have been proposed by independent working groups and experts (e.g., perianal fistulas). To shed light on the actual impact that SR widespread adoption might have in clinical practice, the major results of studies performing a direct comparison between SR and NR are presented. The main characteristics of these are reported in Table 2. In the following paragraphs, organized by district and pathology, a more extensive overview is proposed.

**Table 1:** List of diseases with recommendations for structured abdominal MRI reporting from scientific societies

District	Disease	Purpose	Society	Resources
Upper abdomen	Hepatocellular carcinoma	Detection	ACR	[15]
	Renal masses	Characterization	SAR	[25]
Lower abdomen	Prostate cancer	Detection	ACR, ESUR	[15]
	Bladder cancer	Staging	EAU, JSAR	[35]
	Endometrial cancer^a^	Staging	ESUR	[48]
	Female genital tract congenital anomalies	Detection	ESUR	[52]
	Endometriosis	Detection and disease assessment	ESUR	[55]
Gastrointestinal tract	Rectal cancer	Staging	ESGAR	[64]
	Crohn’s disease	Detection and disease assessment	SAR, AGA	[70]

*ACR* American College of Radiology, *SAR* Society of Abdominal Radiology, *ESUR* European Society of Urogenital Radiology, *EAU* European Association of Urology, *JSAR* Japanese Society of Abdominal Radiology, *ESGAR* European Society of Gastrointestinal and Abdominal Radiology, *AGA* American Gastroenterological Association

^a^Structured reporting template proposed by the Society of Abdominal Radiology available at radreport.org

**Table 2:** Main characteristics of the studies performing a direct comparison between narrative and structured reporting

District	Disease	Authors	Publication year	Study design	Number of MRI exams (lesions)	Number of reports (SR/NR)	Society-endorsed or guidelines-based template	Impact on patient’s outcome	Subjective analysis	Objective analysis
Upper abdomen	Hepatocellular carcinoma	Flusberg et al. [17]	2017	Retrospective	269 (306)	181/125	Yes	N/E	N/P	Number of key imaging findings reported
Lower abdomen	Prostate cancer	Magnetta et al. [29]	2018	Retrospective	300	36/264	Yes	N/E	Urologist and radiologists survey	Number of essential reports components included
		Wetterauer et al. [28]	2019	Retrospective	100	50/50	Yes	N/E	Urologists (4) survey	Ability of correctly plotting the tumor on a map
		Shaish et al. [30]	2018	Retrospective	202 (324)	109/93	Yes	N/E	N/P	Adherence to guidelines scoring system and diagnostic accuracy metrics
	Endometrial cancer	Liu et al. [49]	2019	Retrospective	41	41/41^a^	No	N/E	Gynecologists (5) survey	Number of key imaging findings reported, and time required for reporting
	Uterine fibroids	Franconeri et al. [57]	2017	Retrospective	71	42/29	N/A	N/E	Gynecologists (3) and interventional radiologists (3) survey	Number of key imaging findings reported, and accuracy of measurements
	Endometriosis	Jaramillo‑Cardoso et al. [56]	2019	Retrospective	59 (259)^b^	59/59^a^	No	N/E	N/P	Diagnostic accuracy metrics and interobserver agreement
Gastrointestinal tract	Rectal cancer	Nörenberg et al. [67]	2017	Retrospective	49	49/49^a^	No	N/E	Abdominal surgeons (2) survey	Number of key imaging findings reported
		Sahni et al. [65]	2015	Retrospective	106	43/63	No	N/E	N/P	Quality of report based on key imaging findings reported
		Tersteeg et al. [66]	2018	Retrospective	492	45/447	Yes	N/E	N/P	Number of key imaging findings reported
	Crohn’s disease	Wildman-Tobriner et al. [69]	2016	Retrospective	25	25/25^a^	No	N/E	Referring physicians (3) survey	Number of key imaging findings reported
	Perianal fistulas	Tuncyurek et al. [76]	2018	Retrospective	105	43/62	N/A	N/E	Colorectal surgeons (3) survey	Number of key imaging findings reported

*MRI* magnetic resonance imaging, *SR* structured report, *NR* narrative report, *N/A* not applicable, *N/E* not evaluated, *N/P* not performed

^a^The same MRI exams were reported with both NR and SR

^b^In this case the number in parenthesis indicates compartments explored and not lesions. In the column “subjective analysis”, the number of surveyed referring physicians is reported in parenthesis when available

## Upper abdomen

### Hepatocellular carcinoma

Hepatocellular carcinoma (HCC) is the most common primary liver malignancy and a leading cause of cancer-related deaths [11]. It can be diagnosed noninvasively using contrast-enhanced MRI and the Liver Imaging-Reporting and Data System (LI-RADS) guidelines have been reported to be highly reliable [12–14]. Indeed, they offer recommendations regarding acquisition technique, key imaging findings and provide both a standardized lexicon and a structured reporting template for HCC diagnosis in high-risk patients [15].

MRI structured reporting for HCC diagnosis might offer added value in terms of reproducibility, with evidence of a standardized reporting system predating the release of LI-RADS that showed an intraclass correlation coefficient of 0.80 among 6 radiologists evaluating 100 liver MRI exams [16]. More recently, in a retrospective comparison of a LI-RADS based SR with NR, Flusberg et al. found that final LI-RADS category was more frequently included in the first (98% vs 18%, *p* < 0.001), independently of formal LI-RADS training [17]. Similarly, key imaging findings such as arterial phase hyperenhancement and wash out were found in 98% of SR while not mentioned in 19% and 26% of NR, respectively. However, the study does not provide evidence regarding the impact of SR implementation on patient management and outcome.

### Renal masses

Renal masses are commonly detected as incidental findings on imaging examinations and may prove hard to characterize [18]. Therefore, contrast-enhanced MRI protocols have been designed to establish the most appropriate management strategy for indeterminate renal masses [19–21]. Nevertheless, despite an increase in their incidence and prevalence, content of radiological reports was inconsistent and lacking standardization. Thus, in 2015 a dedicated panel convened by the Society of Abdominal Radiology began working on an evidence-based SR template for renal masses. First, they surveyed both radiologists and urologists to define the key imaging findings to be included in a SR [22]. While the inter-specialty consensus was overall good, the urologists were more likely to prefer the inclusion quantitative data (e.g., nephrometry score or the mass relationship to the renal polar lines) and expressed a preference for clinical management recommendations to not be included in radiology reports [22, 23]. These findings corroborate the need for multidisciplinary engagement to maximize the benefits of SR in terms of communication quality and clinical decision-making. Then, a multicenter, retrospective analysis of radiological reports for indeterminate renal masses was performed and confirmed that some key imaging findings were inconsistently included (such as presence or absence of macroscopic fat -included in 34/236 reports-, size comparisons -included in 111/140 reports- and use of the Bosniak classification for cystic masses -included in 19/35 reports-) with variations across radiologists and practice settings [24]. Finally, evidence-based structured ‘core’ and ‘optional’ templates for reporting indeterminate renal masses were realized using the Delphi technique [25]. Unfortunately, studies formally comparing these SR to conventional NR have not yet been performed. However, given the solid methodological ground on which they are based, with their adoption both improved compliance with reporting key imaging findings and increased referring practitioner satisfaction can be expected.

## Lower abdomen

### Prostate cancer

Since the first version of the Prostate Imaging-Reporting and Data System (PI-RADS) guidelines was released, MRI has become the cornerstone for accurate prostate cancer detection [26]. Notably, the use of standardized reporting for prostate MRI had already been investigated by Quentin and colleagues as a feasible tool to guide MRI-targeted re-biopsy [27]. More recently, Wetterauer et al. retrospectively compared 50 NRs and 50 SRs submitting them to the subjective evaluation of 4 experienced urologists who showed an overall higher satisfaction with structured reporting (mean score on a scale from 1 to 5 equal to 4.5 for SR vs. 2.3 for NR; *p* < 0.01) and declared a significantly inferior need for radiologists’ re-consultation with SR (19% for SR vs 85% for NR; *p* < 0.01) [28]. Furthermore, the urologists were asked to plot the tumor on a prostate diagram using MRI reports information; when using SR, they showed a higher agreement with a radiologist reviewing the MRI scans to plot the same lesions compared to when using NR. However, all NRs in the study were written in 2011 (before PI-RADS implementation) while SRs between 2015 and 2016 (after PI-RADS version 2 was published). Therefore, the results might be due to the increased experience of reporting radiologists and PI-RADS guidelines adoption rather than to the SR itself. With a quality improvement initiative, Magnetta et al. identified report items deemed essential by local referring urologists (e.g., PI-RADS assessment category, findings listed by lesion, prostate-specific antigen density value and low word count) to design and implement a tailored SR template into their dictation software [29]. This intervention lead to an increase in objective quality (measured in terms of essential items reported) and referring urologist satisfaction, who were significantly less incline to contact radiologists for explanation when provided a SR. However, word count did not drop moving from NR to SR and since the same urologists that selected the essential reporting items subsequently performed the subjective quality analysis, the latter could have been partly biased. Moving from a hybrid NR/SR to a full SR based on PI-RADS lexicon and rules, Shaish and colleagues observed a significant improvement of adherence to PI-RADS score rules (88.4% versus 32.9%, *p* < 0.001) [30]. Nevertheless, a survey of specialty societies found that while 54% of the urologists prefer structured reporting for prostate MRI, 53% of the radiologists still indicated a preference towards hybrid NR/SR solutions [31]. Additionally, the preference expressed by radiologists to include clinical management recommendations in their reports is not shared by the urologists, indicating that further interdisciplinary efforts are needed to reach prostate MRI standardized reporting optimization. Finally, beyond prostate cancer detection, prostate MRI standardized reporting might prove useful for local staging as well and scoring system for extraprostatic extension of disease have been proposed [32, 33].

### Bladder cancer

Bladder cancer is associated to high morbidity and mortality, with muscle-invasiveness being a key negative prognostic factor [34]. MRI has been proposed as a feasible diagnostic tool for pre-treatment local staging of the disease and in 2018 the Vesical Imaging-Reporting and Data System (VI-RADS) was released, providing standardized and structured reporting criteria to suggest the likelihood of detrusor muscle invasion [35]. These consensus-based guidelines introduced a five-point probability score derived from T2-weighted, diffusion-weighted and dynamic contrast-enhanced sequence features, and have been endorsed by the Japanese Society of Abdominal Radiology, European Association of Urology, and European Society of Urological Imaging. The validity of VI-RADS has been assessed, with interobserver agreement ranging from good to excellent and an area under the curve for muscle invasion identification as high as 0.94 [36, 37]. Very recently, a meta-analysis performed by Cheng Luo et al. including six studies with 1064 patients confirmed the high diagnostic accuracy of VI-RADS [38]. Furthermore, in a prospective study the VI-RADS score was also found to be a feasible tool to predict the need of repeated transurethral resection of bladder tumor, which is recommended in most high-risk bladder cancer patients not presenting muscle invasion [39]. However, whether the structured format of the MRI report significantly contributes to the success of VI-RADS is still to be verified, as studies formally comparing the use of VI-RADS with SR and NR have not been performed yet.

### Gynecological malignancies: ovarian masses and endometrial cancer

MRI serves as a valuable problem-solving tool for ovarian masses undetermined at ultrasound [40, 41]. In 2013, a standardized reporting system for ovarian mass characterization on MRI was proposed [42]. The validity of this five-category ADNEX MR SCORING system was subsequently verified by independent study groups also confirming that it might lead to the standardization of MRI reporting for ovarian masses [43–45]. Very recently, the MRI working group for the Ovarian-Adnexal Imaging-Reporting and Data System (O-RADS) confirmed with a prospective multicenter study the performance of the ADNEX MR SCORING system (now called the O-RADS MRI score), that represents the current standard in the field [46]. The reproducibility analyses are particularly encouraging, indicating substantial interrater agreement between experienced and junior readers. Therefore, while studies evaluating the role of O-RADS MRI score structured reporting are not yet available, it is fair to assume that the adoption of SR based on the scoring system could be beneficial.

Regarding endometrial cancer, MRI is the imaging modality of choice to perform preoperative disease assessment [47]. In this setting, the European Society of Urogenital Radiology recently released an updated version of their guidelines for endometrial cancer staging with MRI [48]. The working group unanimously agreed on the need for a SR to increase reporting quality and facilitate interdisciplinary communication. A SR template as well as a short mnemonic to summarize the key imaging findings are provided in the guidelines. In a retrospective study, the same radiologist reviewed local staging MRI scans of 41 patients affected by endometrial cancer reporting the images with NR first and using a SR template after 8 weeks and the two reporting strategies were compared [49]. Regarding the objective analysis, the mean number of key features reported was not statistically different between SR and NR (*p* = 0.055). However, the radiologist worked significantly faster when working with the SR (727.22 ± 38.42 s vs. 616.44 ± 60.00 s, *p* = 0.037). Finally, it is worth to mention that a SR template for endometrial cancer staging proposed by the Society of Abdominal Radiology and endorsed by the Template Advisory Library Panel can be found at radreport.org.

### Benign gynecological disorders

There is a plethora of non-oncologic gynecological diseases in which MRI plays an important diagnostic role, and many have worked on MRI structured reporting in this area. In a review, expert radiologists proposed a SR for MRI in patients with infertility [50], while two independent panel of experts defined SR templates for standardizing MRI reporting of chronic pelvic pain [51] and female genital tract congenital anomalies [52] using evidence-based approaches and consensus strategies. Regarding endometriosis, several templates for MRI SR have been proposed in the recent literature [53–55]. In particular, the guidelines released by the Female Pelvic Imaging working group of the European Society of Urogenital Radiology include a dedicated “reporting criteria” section for MRI of endometriosis in which criteria for the diagnosis of endometrial cysts and adhesions as well as the possible locations of deep pelvic endometriosis are defined [55]. However, in a per-location retrospective analysis of 295 compartments (59 patients with surgically confirmed diagnosis), a recent investigation concluded that reader experience may have a greater impact on MRI diagnostic accuracy for deep pelvic endometriosis than reporting style [56].

In the field of benign tumors, the possible role of SR for uterine fibroids MRI has been explored. After the introduction of SR in their department, Franconeri and colleagues retrospectively reported that key features were less consistently included in NR compared to SR (7.3 ± 2.5 vs 1.2 ± 1.5 missing key feature, *p* < 0.0001) [57]. Accordingly, referring practitioners qualitatively described SR as more helpful for treatment planning and easier to understand. Nevertheless, the SR was realized with a multidisciplinary approach which might have affected the qualitative analysis.

### Gastrointestinal tract

#### Rectal cancer

While the prevalence of rectal cancer is rising in developed countries, mortality rate has been declining at least partly due to the added value of MRI in pre- and post-treatment assessment of the disease [58]. Indeed, MRI allows to identify patients that should receive neoadjuvant chemotherapy, aids in surgical approaches customization and can be used for treatment response assessment [59]. With specific regard to rectal cancer MRI staging, a systematic approach to standardize the reporting process was first described in 2008 [60] and shortly after the same working group proposed a SR template including all relevant key imaging findings [61]. Interestingly, in 2014 a synoptic MRI report was successfully developed, and pilot tested in Ontario (Canada), with a 37% adoption rate across the province and leading to a 39% improvement in MRI reports completeness [62]. More recently, the results of scientific societies consensus meetings focused on rectal cancer MRI staging have been released, defining essential items and SR templates for structured reporting [63, 64]. There are some publications suggesting that the use of SR over NR could be beneficial in this field. Sahni et al. found that implementing a SR template and its voluntary use in a radiology department increased the quality of MRI reports for rectal cancer staging, with the percentage of reports including all key imaging features rising from 0 with NR to 41% with SR [65]. However, 30% of all reports produced after the intervention were still unsatisfactory, half of which belonging to the group (20%) that continued to use NR. In the Netherlands, a significant increase in the number of key imaging features reported was observed after the introduction of new guidelines and SR templates for rectal cancer MRI staging (from a median value of 4/10 to 9/10 items, *p* < 0.001) [66]. Noremberg and colleagues evaluated the impact of MRI SR compared to NR in clinical decision-making (surgery vs neoadjuvant radiochemotherapy) for patients with rectal cancer and found that SR allowed a definite choice in 96% of cases vs 60% of NR [67]. Furthermore, SR were considered adequate for surgical planning in 94% of cases while only 38% of NR were judged so by surgeons. However, due to the retrospective design of the study and the lack of a correctness evaluation of reported finding, final conclusions on the clinical impact of SR for rectal cancer MRI staging could not be drawn. Nevertheless, structured reporting appears to be a feasible tool to ensure essential information influencing rectal cancer management are conveyed to referring physicians.

#### Crohn’s disease

Crohn’s disease is a chronic inflammatory bowel disorder which typically involves multiple segments of the gastrointestinal tract, at different time points and with varying degrees of severity [68]. While crucial information can be obtained from clinical and endoscopic evaluation, cross-sectional imaging modalities such as magnetic resonance enterography (MRE), performed with administration contrast mediums both per os and per venam, is often necessary to assess disease status and guide treatment [69]. Recently, The Society of Abdominal Radiology (SAR) Crohn Disease-Focused Panel together with the American Gastroenterological Association (AGA) defined consensus recommendations regarding the use, interpretation and reporting of MRE [70]. In this work, a SR template based on a previous experience is proposed [71]. Rees et al. assessed the inter-reader agreement when using the SAR-AGA recommendations and found a moderate to substantial agreement among five radiologists using structured MRE templates [72]. Since the better results were found for conditions most likely to require intervention (e.g., structuring disease), the authors speculate that SR might influence Crohn’s disease management. Before the SAR-AGA guidelines were released, Wildmann and colleagues compared MRE SR and NR in a population of pediatric patients with Crohn’s disease [69]. They concluded that radiologists objectively detailed nearly two times more key features with structured reporting (a mean of 7.7 ± 2.5 key features for NR compared to 14.0 ± 0.8 for SR, *p* < 0.001). Additionally, the subjective comparative analysis of SR and NR performed by referring physicians revealed a preference towards SR due to higher clarity. However, these retrospective studies included a small number of MRE which were reviewed for the purposes of the analyses and therefore the results might not apply to daily reporting workflow.

#### Perianal fistulas

Perianal fistulas (or fistula-in-ano, FIA) are inflammatory disorders of the anus and perianal soft tissue that can be a cause of substantial morbidity, often requiring surgical intervention [73]. Patients are frequently referred to MRI for the assessment of FIA extent, to verify presence of complications and to confirm the diagnosis in challenging cases. Recently, SR templates for MRI of FIA have been published, sharing similar key imaging features (e.g., FIA type, extension and accessory tracts, presence of abscess) and highlighting the importance of clock-face annotation to indicate position [74, 75]. In both cases, the authors emphasized that SR templates could optimize patient management through clear and complete communication of key imaging findings while reducing reporting time and making MRI studies easier to compare. Performing a retrospective comparison, Tuncyurek et al. confirmed that a disease-specific SR is superior to conventional reporting for FIA MRI [76]. Specifically, an objective analysis found that in NR more key features were absent than in SR (respectively 6.3 ± 1.8 vs 0.3 ± 0.9, *p* ≤ 0.001). In the subjective surgeons’ judgment, SR was also deemed to be clearer compared to NR. Of note, the authors highlight the importance of education on top of report standardization since radiologists that kept using the NR after SR introduction began including more key imaging features too.

## Discussion

Physicians rely on a written interpretation of MRI findings to assist them in patient management while patients demand clearer, more accessible radiology reports [2]. They are the most important element on which our work is assessed, and NR have been often criticized as a vestige of the past and at risk of being inconsistent and therefore unreliable [77]. While possibly offering a solution to this problem, the implementation of SRs is a complex and debated issue [78]. There are recognized limitations of SR, such as the risk of excessive simplification and template layout rigidity as well as poor user compliance [79]. Nevertheless, as described previously significant efforts have been made by scientific societies and panels of experts towards the definition of common SR templates for abdominal MRI in various settings. Their implementation in radiology departments with high adoption rates might not be overly complicated and tailoring on departmental specific characteristics and in accordance with local referring physicians is possible and should be considered [80]. Herts et al. propose to realize a system-specific report and then disease-specific report templates by expanding on the first [10]. Such an approach could allow SR to appear less rigid and be more easily embraced. However, this might still prove challenging and SR templates might not be able to meet every reporting need as they appear best suited for specific conditions rather than complex patients [78]. Additionally, many among the SR templates presented in this review have only been released very recently. Another issue that emerges from this review of the literature is the lack of studies proving a significant benefit for patient outcome associated to SR. Currently available data from formal comparisons between NR and SR are based on subjective (perceived quality questionnaires) and objective (key imaging findings reported) evaluations performed retrospectively, which do not provide the desirable level of evidence to confirm the urgency for a widespread adoption of structured reporting. It is possible that the same advantages of SR use could be obtained by proper education of subspecialty radiologists and promoting the use of standardized lexicon and validated scoring systems without the need for actual structuring of reports, therefore defending the autonomy of radiologists [81]. It could also be argued that multidisciplinary meetings and radiologist consultations might be a different feasible solution to increase the value and impact of our work [82]. If prospective studies designed to clarify these issues will not be performed, there is a risk that the debate on reporting strategies will not be settled, limiting SR implementation. Finally, there are diseases in which the possible advantage of MRI SR remains largely unexplored (e.g., pancreatic cystic lesions, adrenal masses, biliary duct pathology, outlet obstruction).

In conclusion, the assessment of certain abdominal disorders by means of MRI might benefit from the adoption of SR over NR in terms of higher reporting quality and referring physician’s satisfaction. Current evidence suggests that SR tends to be more comprehensive, including a higher number of key imaging findings compared to NR. A multitude of templates is already available, but further and stronger evidence confirming their value is needed, especially regarding patient’s outcome improvement.

## Abbreviations

MRI: Magnetic resonance imaging
NR: Narrative report
SR: Structured report
HCC: Hepatocellular carcinoma (HCC)
LI-RADS: Liver Imaging-Reporting and Data System
PI-RADS: Prostate Imaging-Reporting and Data System
O-RADS: Ovarian-Adnexal Imaging-Reporting and Data System
VI-RADS: Vesical Imaging-Reporting and Data System
MRE: Magnetic resonance enterography
SAR: Society of Abdominal Radiology
AGA: American Gastroenterological Association
FIA: Fistula-in-ano
